# 2234. Ambulatory Prescribing Trends of Azithromycin and Systemic Glucocorticoids in Patients Diagnosed with *COVID-19*: A Large Healthcare Network Analysis in Massachusetts

**DOI:** 10.1093/ofid/ofad500.1856

**Published:** 2023-11-27

**Authors:** Shamsuddin Anwar, Maureen Campion, Kap Sum Foong, Shira Doron, Gabriela Andujar Vazquez

**Affiliations:** Tufts Medical Center, Boston, Massachusetts; Tufts Medical Center, Boston, Massachusetts; Tuft Medical Center, Boston, Massachusetts; Tufts Medical Center, Boston, Massachusetts; Tufts Medical Center, Boston, Massachusetts

## Abstract

**Background:**

Data do not support the use of azithromycin (AZ) and systemic glucocorticoids (GCS) for nonhospitalized adult patients with COVID-19. However, these two medications remain commonly prescribed in the ambulatory setting. We aimed to evaluate prescribing trends of AZ and GCS in patients diagnosed with COVID 19 at ambulatory clinics from a large Boston area healthcare network.

**Methods:**

We performed a retrospective cohort study of all ambulatory clinics (i.e., primary care and specialty) affiliated with Tufts Medicine healthcare network from April 2020 through May 2022. These clinics were categorized into academic medical center-based (AMCB) clinic or community-based (CB) practices. We queried the system informatics database to identify all outpatient COVID-19 encounters using ICD-10-CM diagnosis code (U07.1). Demographics, treatment, and prescriber data were extracted. We performed χ2 to compare categorical variables.

**Results:**

A total of 44,369 outpatient COVID-19 encounters were identified; 40.7% occurred in AMCB clinics and 59.3% in CB clinics. Within these encounters, 1,798 (4.1%) and 3,561 (8.0%) had prescriptions for AZ and GCS, respectively. Prescriptions varied by month, with a higher rate of prescriptions during SARS COV 2 variant surges (i.e., Winter period 2020-2021, Delta, Omicron)(Figure 1). The rates of AZ (3.6% vs 4.4%, p< .001) was significantly higher in CB clinics compared to AMCB while rates of GCS (9.5% vs. 7.0%, p< .001) prescriptions were significantly higher in AMCB.

Prescription Trends For Azithromycin And Systemic Glucocorticoids From May 2020 to April 2022

**Figure:**
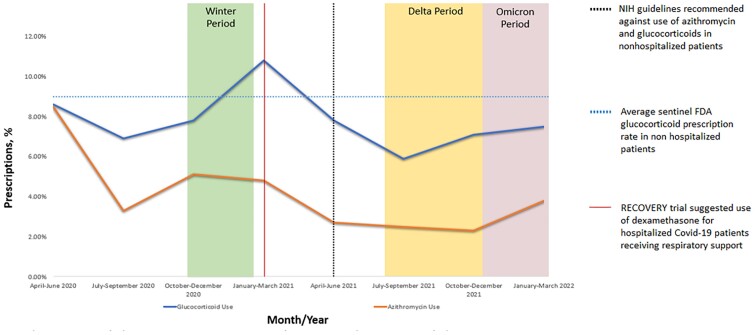

**Conclusion:**

Despite national guidelines recommending against the use of AZ and GCS for nonhospitalized adult patients with COVID-19, we observed their use, and noted higher rates of prescription during SARS COV-2 variant surges. While the overall prescription rates for AZ and GCS were similar to national trends, we found use to be significantly varied in CB and AMCB clinics. Our study highlights opportunities for targeted intervention to reduce inappropriate prescribing practices for patients diagnosed with COVID-19 in the ambulatory setting.

**Disclosures:**

**Maureen Campion, PharmD, BCIDP**, Shinoigi: Speaker

